# Disruption of mitochondrial homeostasis and permeability transition pore opening in OPA1 iPSC-derived retinal ganglion cells

**DOI:** 10.1186/s40478-025-01942-z

**Published:** 2025-02-13

**Authors:** Michael Whitehead, Joshua P. Harvey, Paul E. Sladen, Giada Becchi, Kritarth Singh, Yujiao Jennifer Sun, Thomas Burgoyne, Michael R. Duchen, Patrick Yu-Wai-Man, Michael E. Cheetham

**Affiliations:** 1 UCL Institute of Ophthalmology, 11-43 Bath Street, London, EC1V 9EL UK; 2 Department of Cell and Developmental Biology and Consortium for Mitochondrial Research, UCL, Gower St, London, WC1E 6BT UK; 3 John van Geest Centre for Brain Repair, University of Cambridge, Cambridge, CB2 0PY UK; 4 MRC Mitochondrial Biology Unit, Department of Clinical Neurosciences, University of Cambridge, Cambridge, CB2 0XY UK; 5 Cambridge Eye Unit, Addenbrooke’s Hospital, Cambridge University Hospitals NHS Foundation Trust, Cambridge, CB2 0QQ UK; 6 Moorfields Eye Hospital NHS Foundation Trust, London, EC1V 2PD UK

**Keywords:** Dominant optic atrophy, OPA1, iPSCs, Retinal ganglion cells, Neurodegeneration, Mitochondrial networks, Calcium homeostasis

## Abstract

Dominant optic atrophy (DOA) is the most common inherited optic neuropathy, characterised by the selective loss of retinal ganglion cells (RGCs). Over 60% of DOA cases are caused by pathogenic variants in the *OPA1* gene, which encodes a dynamin-related GTPase protein. OPA1 plays a key role in the maintenance of the mitochondrial network, mitochondrial DNA integrity and bioenergetic function. However, our current understanding of how OPA1 dysfunction contributes to vision loss in DOA patients has been limited by access to patient-derived RGCs. Here, we used induced pluripotent stem cell (iPSC)-RGCs to study how OPA1 dysfunction affects cellular homeostasis in human RGCs. iPSCs derived from a DOA+ patient with the OPA1 R445H variant and isogenic CRISPR-Cas9-corrected iPSCs were differentiated to iPSC-RGCs. Defects in mitochondrial networks and increased levels of reactive oxygen species were observed in iPSC-RGCs carrying OPA1 R445H. Ultrastructural analyses also revealed changes in mitochondrial shape and cristae structure, with decreased endoplasmic reticulum (ER): mitochondrial contact length in DOA iPSC-RGCs. Mitochondrial membrane potential was reduced and its maintenance was also impaired following inhibition of the F1Fo-ATP synthase with oligomycin, suggesting that mitochondrial membrane potential is maintained in DOA iPSC-RGCs through reversal of the ATP synthase and ATP hydrolysis. These impairments in mitochondrial structure and function were associated with defects in cytosolic calcium buffering following ER calcium release and store-operated calcium entry, and following stimulation with the excitatory neurotransmitter glutamate. In response to mitochondrial calcium overload, DOA iPSC-RGCs exhibited increased sensitivity to opening of the mitochondrial permeability transition pore. These data reveal novel aspects of DOA pathogenesis in R445H patient-derived RGCs. The findings suggest a mechanism in which primary defects in mitochondrial network dynamics disrupt core mitochondrial functions, including bioenergetics, calcium homeostasis, and opening of the permeability transition pore, which may contribute to vision loss in DOA patients.

**Supplementary Information:**

The online version contains supplementary material available at 10.1186/s40478-025-01942-z.

## Introduction

Dominant optic atrophy (DOA) is the most common inherited optic neuropathy, with an estimated minimum prevalence of 1 in 25,000 [1]. Clinically, DOA usually presents in the first two decades of life, characterised by bilateral progressive loss of central vision, dyschromatopsia and the development of optic disc pallor [2]. Optical coherence tomography (OCT) studies have demonstrated thinning of the retinal nerve fibre layer (RNFL), in particular the papillomacular bundle, highlighting the preferential loss of retinal ganglion cells (RGCs) in DOA pathogenesis [3].

Around 60% of people with DOA have pathogenic *OPA1* variants [4]. OPA1 is a dynamin-related GTPase protein that localises to the inner mitochondrial membrane (IMM) [5]. OPA1 is ubiquitously expressed, and whilst loss of OPA1 function appears to primarily affect RGC function in DOA, approximately 20% of DOA patients exhibit a more severe syndromic ‘DOA+’ phenotype, characterised by multisystem neurodegeneration. These patients exhibit a broad range of neurological defects, including sensorineural hearing loss, ataxia, peripheral neuropathy, and myopathy [2, 6]. At the molecular level, DOA+ is often associated with missense variants in the GTPase domain, such as R445H, and this could be due to a dominant negative effect in which dysfunctional, pathogenic OPA1 variants impair WT OPA1 protein function, driving a more severe clinical phenotype [2].

OPA1 maintains mitochondrial network dynamics by facilitating fusion of the IMM, possibly through a mechanism involving cardiolipin [7]. As such, loss of OPA1 function has been associated with defects in mitochondrial network morphology [8, 9]. OPA1 also maintains mitochondrial cristae shape, and loss of OPA1 function has been correlated with aberrant cristae architecture [10] and cytochrome *c* release [11], highlighting a possible pathogenic mechanism underpinning RGC cell death in DOA. Reduced OPA1 function causes impaired function of the electron transport chain (ETC), with reduced oxygen consumption [12–14], and a reduced mitochondrial membrane potential (MMP) [11, 15, 16]. These bioenergetic defects may indirectly sensitise RGCs to cell death in DOA via autophagic [17, 18] or excitotoxic [19, 20] mechanisms, possibly via ATP depletion. OPA1 also participates in the maintenance of mitochondrial DNA (mtDNA) integrity, with a postulated role in anchoring mtDNA molecules to the IMM [21], however, distinct effects of OPA1 dysfunction on mtDNA have been reported across different tissues/cell types [22, 23].

OPA1 dysfunction may impact calcium homeostasis in a cell type-dependent manner. Reports that OPA1 downregulation enhanced mitochondrial calcium uptake in patient-derived fibroblasts [24] conflicted with reports that siRNA-mediated *OPA1* knock-down reduced mitochondrial calcium uptake in HeLa cells [19]. Whilst pro-opiomelanocortin neurons in which OPA1 protein levels were experimentally knocked down displayed unaltered cytosolic, but attenuated mitochondrial calcium transients [25], siRNA-mediated OPA1 knockdown in rat RGCs led to delayed calcium deregulation (DCD) in response to excitotoxic stress, an event that preceded cell death [19]. Furthermore, reduced OPA1 function increased basal cytosolic calcium levels, leading to cell death in mouse RGCs and *Caenorhabditis elegans* GABAergic motor neurons transfected with plasmids containing pathogenic variants of *OPA1* [26]. These findings highlight the importance of studying the effect of OPA1 dysfunction on calcium homeostasis in human RGCs to better understand pathophysiological mechanisms in DOA patients.

Here, we used OPA1 R445H patient-derived and CRISPR-Cas9-corrected iPSC-RGCs to investigate the hypotheses that OPA1 dysfunction leads to changes in mitochondrial structure, network morphology and reactive oxygen species in RGCs. The potential effect on calcium homeostasis was also investigated, as well as the mechanism of maintenance of the MMP, and the threshold for opening of the mitochondrial permeability transition pore (mPTP) in response to mitochondrial calcium overload as a potential factor in RGC cell death in DOA.

## Materials and methods

### Generation, maintenance and differentiation of iPSC lines

Male patient-derived dermal fibroblasts carrying the c.1334G >A (p. R445H) substitution were reprogrammed to iPSCs via nucleofection of episomal reprogramming vectors, and isogenic controls were created using CRISPR-Cas9 gene editing, as described previously [27]. iPSCs were maintained on Geltrex-coated plates in mTeSR media (Stem Cell Technologies) at 37 °C 5% CO_2_. For passaging, iPSC colonies were manually dissociated twice weekly.

One patient and one corrected iPSC line (between passage number 21–35) were differentiated to iPSC-RGCs as previously described using a 42 day (D42) directed differentiation protocol [12]. Briefly, iPSCs were plated at 500,000 cells/well on a Matrigel (Corning)-coated 6WP, in mTeSR media. Media was changed the next day, and subsequently changed every two days, with a 1:1 mixed DMEM: F12/Neurobasal (Gibco) basal media supplemented with 1% N2 and 2% B27 (Gibco). N2B27 media was supplemented with the following compounds on the indicated days. For D1-6, dorsomorphin (1 µM, Stratech) and IDE2 (2.5 µM, Peprotech), for D1-D10, Nicotinamide (10 mM, Sigma), for D1-D35, Forskolin (25 µM, Peprotech), and for D17 to D30, DAPT (10 µM, Abcam), for D35-D42, CultureOne (Gibco) to inhibit any mitotic cell division. All experiments were replicated across at least three independent differentiations, see figure legends for specific details. Successful differentiation to iPSC-RGCs was monitored by morphological assessment throughout the differentiation and parallel qPCR and immunocytochemistry for RGC markers, an example is shown in the supplementary material (Supplementary Figure S1).

### Quantitative reverse transcription polymerase chain reaction (qPCR)

After washing with PBS, RNA was extracted from iPSC-RGC cultures with RNeasy Mini Kit (Qiagen), and 500ng per sample was converted to cDNA with the Tetro cDNA Synthesis Kit (Tetro). cDNA samples were diluted 1:10 in nuclease-free water, and mixed with 10 µL 2x LabTaq (Labtech), 0.8uL forward and reverse primers (10 μm), and topped up to 20 uL total volume with nuclease-free water. Samples were run in a thermocycler (QuantStudio 6) according to the following conditions: 2 min at 95 °C, then 40 cycles of 1s at 95 °C, 25s at 60 °C. RGC markers *BRN3B*, *SNCG* and *ISL1* were normalised to *ACTIN* and *GAPDH* loading controls, quantification of RNA expression was performed using the ΔΔCt method. The primers were as previously described [12].

### Immunocytochemistry

To measure mitochondrial network dynamics, 25 nM Mitotracker Orange (Thermo Fisher Scientific) was loaded for 30 min at 37 °C 5% CO_2_ in recording buffer (150 mM NaCl, 4.25 mM KCl, 4 mM NaHCO_3_, 1.25 mM NaH_2_PO_4_, 1.2 mM CaCl_2_, 1.2 mM MgCl_2_, 10 mM D-glucose, and 10 mM HEPES at pH 7.4). Neurons were washed, then fixed in 4% paraformaldehyde for 15 min. When assessing network dynamics in neuronal cell bodies, cells were washed and immediately mounted onto a glass slide with Fluoromount (Dako). Mitotracker Orange was excited at 555 nm and a ≥ 580 nm emission filter was used. Leica LAS X software was used to optimise image acquisition parameters (e.g. optimal number of steps in a stack according to pinhole diameter, magnification etc.), and Leica Lightning software was used for image deconvolution, using a refractive index of 1.52. ImageJ plugin MINA Version 3 (https://imagej.net/plugins/mina) was used for quantification. Mitochondrial footprint = volume of the mitochondrial signal; branch length mean = mean length of all the lines used to represent the mitochondrial structure; summed branch length mean = sum of all branch lengths divided by the number of independent skeletons; network branches mean = mean number of attached lines used to represent each structure. To assay mitochondrial length/distribution in neurites, after fixation and washing, neurons were incubated in blocking solution (10% normal donkey serum, 1% bovine serum albumin) for 1 h, then incubated overnight at 4 °C with rabbit anti-tubulin beta 3 (TUBB3) antibodies (Abcam) in 50% blocking buffer diluted in PBS. Anti-rabbit AlexaFluor 488 secondary antibodies (Thermo Fisher Scientific) were incubated for 2 h at room temperature, before mounting the cells onto a glass slide. Images were acquired on a Stellaris 8 confocal microscope equipped with a 40x oil objective.

### Detection of reactive oxygen species

iPSC-RGCs were plated in 96 well black-walled plates (Thermo Fisher Scientific). After washing with recording buffer, neurons were stained with 5 µM dihydroethidium (DHE; Thermo Fisher Scientific) or 5 µM MitoSOX (Thermo Fisher Scientific) for 30 min at 37 °C 5% CO_2_ in recording buffer. Assessment of DHE/MitoSOX fluorescence was performed on a Cytation 10 microplate reader (Agilent). DHE was excited at 518 +/- 20 nm, emission 606 +/- 20 nm. MitoSOX was excited at 535 +/- 20 nm, emission 585 +/- 20 nm. Hoechst dye was added at 10 µg/mL for 10 min at the end of the experiment to normalise DHE/MitoSOX signals to total cell number. Hoechst was excited at 350 +/- 20 nm, emission 450 +/- 20 nm.

### Western blotting

iPSC-RGCs were lysed in RIPA buffer (1% NP-40, 20 mM Tris-HCl, 5 mM sodium pyrophosphate, 5 mM EDTA) with 2% protease inhibitor cocktail (Sigma Aldrich). 5 µg of protein was loaded into 10% polyacrylamide gels, resolved at 80 V for 2 h, then transferred onto nitrocellulose membranes at 90 V for 90 min. Membranes were blocked with 5% milk powder (diluted in PBS 0.2% Tween-20) (Sigma Aldrich) for 1 h, then incubated with primary antibodies (Santa Cruz sc-17767 mouse anti-SOD1; 1:100 dilution, Abcam 18207 rabbit anti-TUBB3 1:5,000 dilution, or Proteintech 60004-1 mouse anti-GAPDH 1:8,000 dilution) overnight at 4 °C. Membranes were incubated with HRP-conjugated goat anti-mouse secondaries for 1 h at room temperature, bands were visualised with ECL Clarity Substrate (Biorad) and a Chemidoc imaging system (Biorad), using multiple exposures to avoid saturating band intensity. Quantification of band intensity was performed in ImageLab (BioRad) and SOD1 immunoreactivity normalised against reference proteins TUBB3 or GAPDH. Uncropped blots are available in supplementary material (Supplementary Figure S2).

### Live cell confocal imaging experiments

For live imaging assessments, iPSC-RGCs were plated on 35 mm Fluorodishes (World Precision Instruments). All live imaging experiments were performed with cells incubated in recording buffer, with two exceptions: for glutamate stimulation, MgCl_2_ was removed to maximise activation of N-methyl D-aspartate (NMDA) receptors; for ER calcium release/SOCE, cells were kept in calcium-free recording buffer until the introduction of 1.2 mM CaCl_2_ at the end of the experiment. iPSC-RGCs were maintained at 37 °C 5% CO_2_ throughout image acquisition. All confocal imaging was performed on a Stellaris 8 microscope equipped with a 20x dry or 40x oil objective. Regions of interest (ROIs) were selected based on the cells having a neuronal morphology. Example ROIs are available in the supplementary material (Supplementary Figure S3).

For measurement of cytosolic calcium levels, iPSC-RGCs were loaded with 1.5 µM Fluo4 AM (Thermo Fisher Scientific) for 30 min at 37 °C 5% CO_2,_ then washed twice to remove any residual Fluo4 AM. iPSC-RGCs were exposed to 1 µM thapsigargin (Selleck Chemicals), 1.2 mM CaCl_2_, 5 µM glutamate (Sigma Aldrich), or 10 µM ionomycin (Sigma Aldrich) at the indicated time points. To assay MMP, iPSC-RGCs were loaded with 5 nM tetramethylrhodamine ethyl ester (TMRE; Thermo Fisher Scientific) for 30 min at 37 °C 5% CO_2_. 5 nM TMRE was kept in the recording buffer throughout the experiment. iPSC-RGCs were incubated with 1.5 µM oligomycin (Sigma Aldrich) or ascending doses (2.5–17.5 µM; 2.5 µM each step) of ferutinin (Sigma Aldrich) at the indicated time points. Fluo4 was excited at 488 nm, collecting light longer than 520 nm, whilst TMRE was excited at 555 nm, collecting light longer than 580 nm. These experiments were performed at 0.1% laser strength to minimise the effect of photobleaching/oxidative damage. Analysis of Fluo4/TMRE fluorescence was performed in ImageJ.

### Transmission electron microscopy imaging

Cells were fixed by adding 4% PFA and 4% glutaraldehyde in 0.1 M cacodylate buffer at pH 7.4 to the cell culture media at 1:1 and left for 1 h at room temperature. The cells were washed in 0.1 M cacodylate buffer before incubating in 1% osmium tetroxide and 1.5% potassium ferrocyanide in distilled water for 1 h in the dark at 4 °C. En bloc staining was performed by incubating the cells in UA-Zero (Agar Scientific, Stansted, UK) for 1 h in the dark at room temperature. Subsequently, the cells were dehydrated in increasing concentrations of ethanol (70%, 90%, and 100%) followed by a mixture of propylene oxide: epon (1:1) overnight at room temperature. The propylene oxide: epon was replaced with two changes of epon every 3 h at room temperature before embedding in epon overnight at 60 °C. 100 nm sections were cut and imaged on a JEOL 1400Plus EM (JEOL ltd, Tokyo, Japan) fitted with an Advanced Microscopy Technologies (AMT) NanoSprint12 (AMT Imaging Direct, Woburn, MA, USA) camera. Analysis of TEM imaging was performed in ImageJ. Mito/cristae circularity = 4π*area/perimeter^2.

## Results

### Disturbed mitochondrial structures in OPA1 R445H iPSC-RGCs

**Fig. 1:**
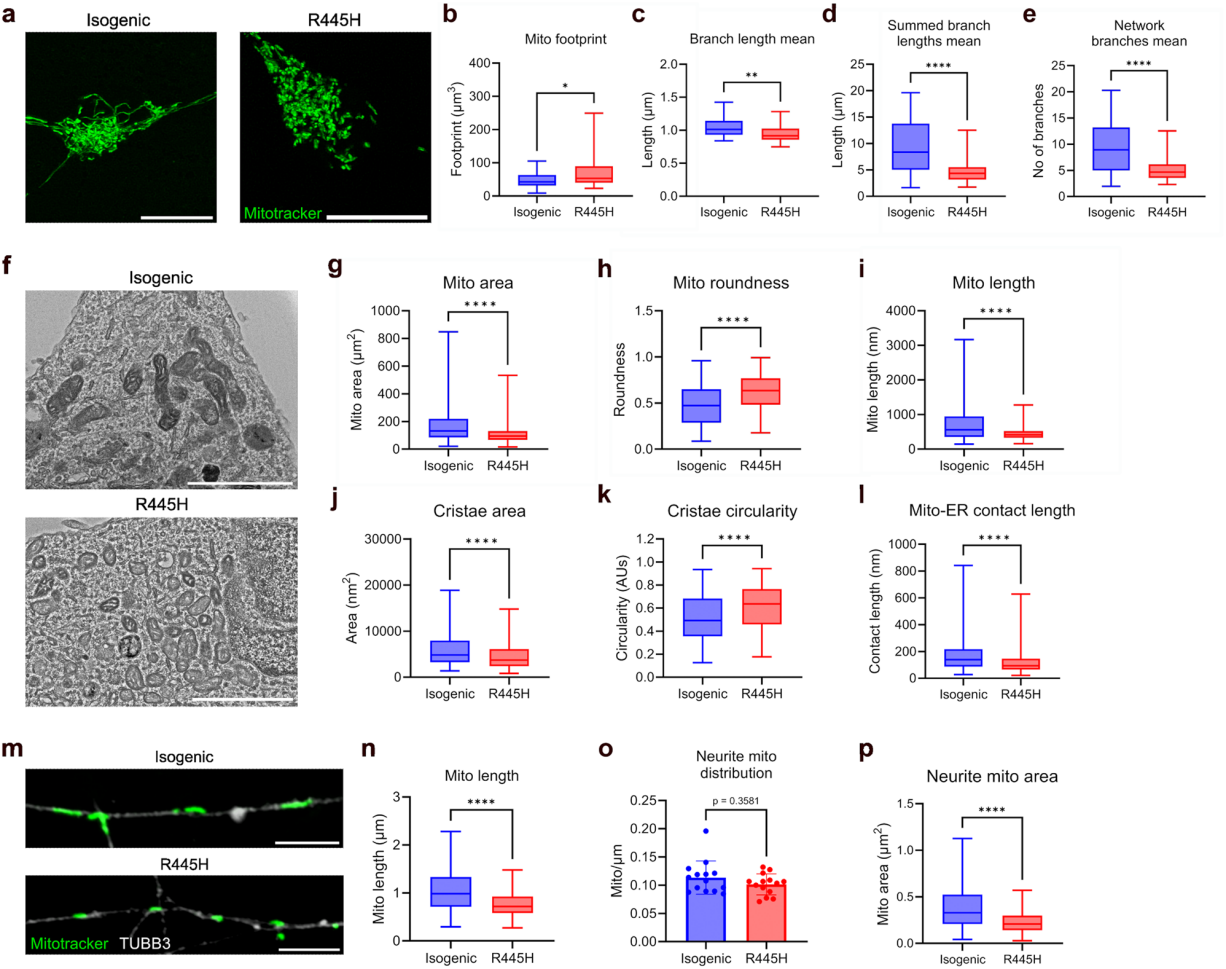
Defects in mitochondrial structure in OPA1 R445H iPSC-RGCs. (**a**) Representative images of the mitochondrial network in R445H and isogenic control neurons stained with Mitotracker dye, deconvoluted with Lightning software. Scale bar, 10 μm. (b-e) MINA analysis of mitochondrial networks. Isogenic, *n* = 37; R445H, *n* = 34 cells, sampled from three independent differentiations. (**b**) and (**c**), ** = *p* < 0.01, Mann-Whitney tests, (**d**) and (**e**), *** = *p* > 0.001, Welch’s t-tests. Box plots show median (middle line), 25th-75th percentile (box) and min/max values (whiskers). (**f**) Representative TEM images of mitochondrial structures in isogenic control and R445H iPSC-RGCs. Scale bar, 2000 nm. (**g**-**i**) Quantification of mitochondrial area, roundness, and length, performed in ImageJ. Isogenic, *n* = 483; R445H, *n* = 510 ROIs, sampled from three independent differentiations. **** = *p* < 0.0001, Mann-Whitney tests. Box plots show median (middle line), 25th-75th percentile (box) and min/max values (whiskers). (**j** & **k**) Quantification of mitochondrial cristae shape, performed in ImageJ. Isogenic, *n* = 292; R445H *n* = 280 ROIs sampled from three independent differentiations. **** = *p* < 0.0001, Mann-Whitney tests. Box plots show median (middle line), 25th-75th percentile (box) and min/max values (whiskers). (**l**) Quantification of mitochondria: ER contact sites, performed in ImageJ. Isogenic, *n* = 350; R445H *n* = 419 ROIs sampled from three independent differentiations. **** = *p* < 0.0001, Mann-Whitney tests. Box plots show median (middle line), 25th-75th percentile (box) and min/max values (whiskers). (**m**) Representative images of Mitotracker-stained mitochondria in TUBB3 + neurites. Scale bar, 5 μm. (**n**) Quantification of mitochondria length, performed in ImageJ. Isogenic, *n* = 1,241 ROIs; R445H, *n* = 625 ROIs, sampled from three independent differentiations. **** = *p* < 0.0001, Mann-Whitney test. Box plot shows median (middle line), 25th-75th percentile (box) and min/max values (whiskers). (**o**) Quantification of mitochondria distribution in TUJ1 + neurites. Isogenic/R445H, *n* = 14 images acquired from three independent differentiations. No significant difference observed between genotypes, *p* = 0.358, Mann-Whitney test. Scatter plot shows the mean values +/- SD. (**p**) Quantification of mitochondrial area in TUBB3 + neurites. Isogenic, *n* = 1,209 ROIs; R445H, *n* = 590 ROIs, sampled from three independent differentiations. **** = *p* < 0.0001, Mann-Whitney test. Box plot shows median (middle line), 25th-75th percentile (box) and min/max values (whiskers)

OPA1 participates in the maintenance of mitochondrial network morphology [10]. We sought to establish whether OPA1 dysfunction was associated with changes in the structure of the mitochondrial network in D42 iPSC-RGC cell bodies, and mitochondrial length in D42 iPSC-RGC neurites, using Mitotracker dyes to delineate mitochondrial structure (Fig. 1a). Neuronal soma with the R445H pathogenic OPA1 variant (derived from a DOA + patient) displayed a 1.5-fold increased mitochondrial footprint compared to corrected isogenic controls (Fig. 1b). Conversely, mean branch length, mean summed branch length, and mean network branches were decreased in RGCs carrying the R445H pathogenic variant compared to isogenic iPSC-RGCs (Fig. 1c-e), suggesting fragmentation of mitochondrial networks is associated with OPA1 dysfunction in human RGCs.

To extend these data, mitochondrial ultrastructure was assessed with transmission electron microscopy (TEM) imaging (Fig. 1f). The area and length of individual mitochondria were reduced, whilst roundness was increased in R445H iPSC-RGCs compared to isogenic controls (Fig. 1g-i). These data again support fragmentation of mitochondrial structures in cells with pathogenic *OPA1* variants. Mitochondrial cristae architecture was also altered between R445H and isogenic control iPSC-RGCs. In R445H neurons, mitochondrial cristae more commonly displayed a vesicular architecture compared to isogenic control cells. In support of this, cristae area was reduced in R445H cells, whilst circularity was increased compared to isogenic control samples (Fig. 1j & k).

We then tested if OPA1 dysfunction was associated with changes in mitochondria: ER membrane contact (MERC) length in our iPSC-RGC model, reasoning that similar defects have been observed across a range of neuropathologies [28], and observed that contact lengths were reduced in R445H vs. isogenic control iPSC-RGCs (Fig. 1l).

iPSC-RGC neurite mitochondrial morphology (Fig. 1m) was investigated, and it was observed that R445H neurite mitochondrial length was decreased compared to isogenic control cells (Fig. 1n). A statistically significant change was not observed between genotypes in the distribution of mitochondria in iPSC-RGC neurites (Fig. 1o); however, mitochondrial area in TUBB3 + neurites was significantly lower in R445H iPSC-RGCs compared to isogenic controls (Fig. 1p). Ultrastructural analyses by TEM, revealed that the morphology of the OPA1 R445H neurite mitochondria was similar to the isogenic control neurite mitochondria, unlike in the neuronal soma (Supplementary Figure S4). The neurite mitochondria were rod like with sparse tubular cristae in both genotypes; however, the R445H neurite mitochondria were generally shorter than the isogenic, consistent with the immunofluorescence data. This suggests there might be some quality control of the mitochondria that are allowed to traffic to the neurites, or that the physical constraints and trafficking machinery within the neurite contribute to mitochondrial shape. Consistent with the role of OPA1 as a pro-fusion factor, collectively these data demonstrate fragmentation of mitochondrial networks and highlight potential changes in mitochondrial cristae and MERCs in DOA iPSC-RGCs.

### Oxidative stress and MMP defects in OPA1 R445H iPSC-RGCs

**Fig. 2:**
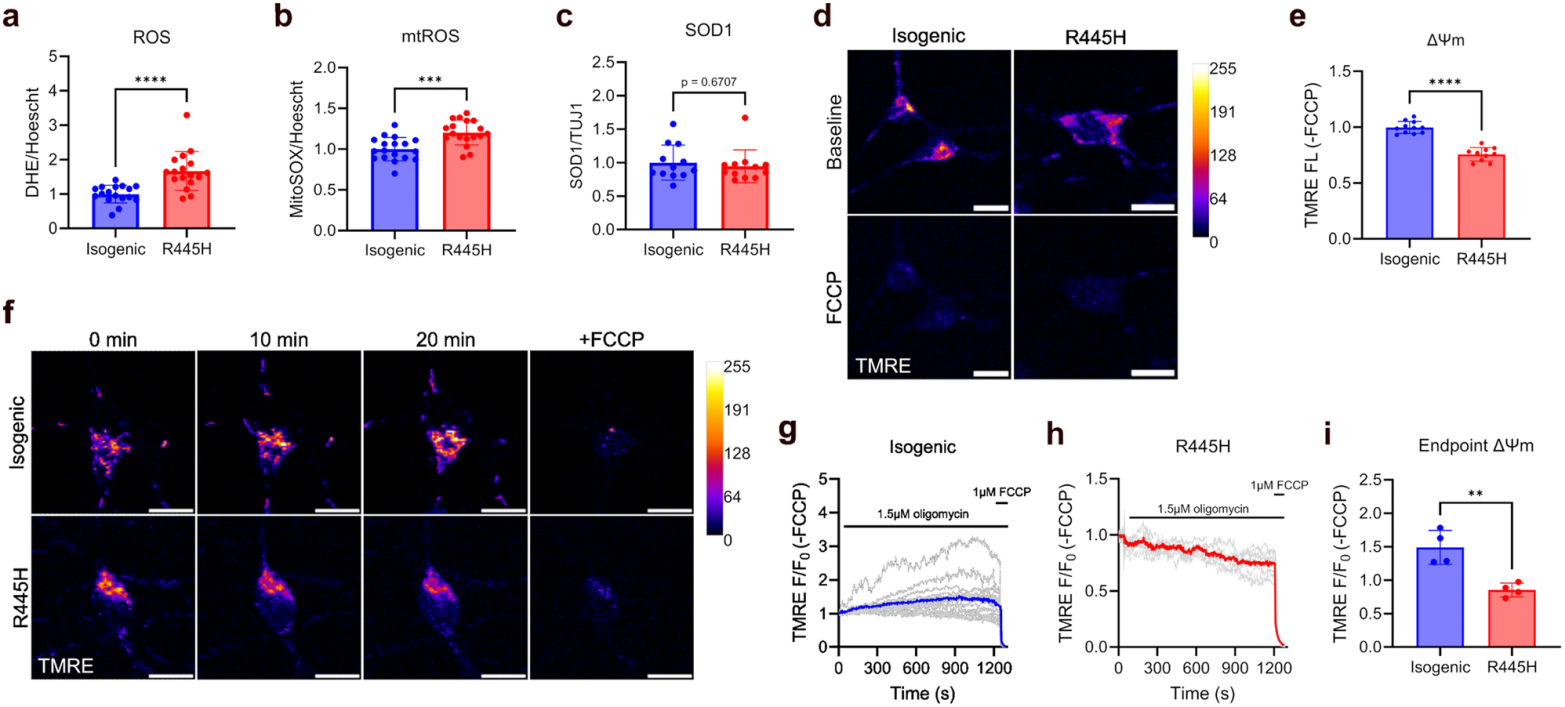
Oxidative stress and MMP defects in OPA1 R445H iPSC-RGCs. (**a**) DHE fluorescence signal normalised to cell number (Hoechst fluorescence). Isogenic/R445H, *n* = 18 wells sampled from three independent differentiations. **** = *p* < 0.0001, Mann-Whitney test. Scatter plot shows the mean values +/- SD. (**b**) MitoSOX fluorescence signal normalised to cell number (Hoechst fluorescence). Isogenic/R445H, *n* = 18 wells sampled from three independent differentiations. *** = *p* < 0.001, unpaired t-test. Scatter plot shows the mean values +/- SD. (**c**) Quantification of SOD1 Western blots. Isogenic/R445H, *n* = 12 sampled from three independent differentiations. No significant differences were observed between genotypes, *p* = 0.671, Mann-Whitney test. Scatter plot shows the mean values +/- SD. Western blot data is available in Supplementary Fig. 1. (**d**) Representative live cell confocal images of MMP in TMRE-stained isogenic control and R445H iPSC-RGCs at baseline and after FCCP treatment. Scale bar, 10 μm. (**e**) Quantification of MMP expressed as the fold-change in TMRE fluorescence relative to isogenic control samples. FCCP was used as a negative staining control to subtract non-mitochondrial TMRE fluorescence. Isogenic, *n* = 11; R445H, *n* = 10 images sampled from three independent differentiations. **** = *p* < 0.0001, unpaired t-test. Scatter plot shows the mean values +/- SD. (**f**) Representative live cell confocal images of TMRE-stained isogenic control and R445H iPSC-RGCs at baseline, 10 and 20 min after addition of 1.5 µM oligomycin, and following FCCP treatment. Scale bar, 10 μm. (**g** & **h**) Quantification of fold-change in TMRE fluorescence over time in isogenic control and R445H neurons. Fluorescence intensity was normalised to baseline values (F_0_). 1.5 µM oligomycin and 1 µM FCCP were added at the indicated time points. FCCP was used as a negative staining control to subtract non-mitochondrial TMRE fluorescence. Grey lines show individual cells TMRE fluorescence intensity from a representative experiment, blue/red lines the mean average normalised TMRE fluorescence intensity. (**i**) Quantification of endpoint TMRE fluorescence in isogenic control and R445H iPSC-RGCs prior to FCCP addition. Isogenic/R445H, *n* = 4 images acquired from three independent differentiations. ** = *p* < 0.01, unpaired t-test. Scatter plot shows the mean values +/- SD

We previously reported a bioenergetic defect in iPSC-RGCs harbouring pathogenic *OPA1* variants [12]. Mitochondrial dysfunction in neurodegenerative disease is often associated with increased generation of reactive oxygen species (ROS) [29], in particular mitochondrial superoxide production arising from ETC dysfunction [30]. Oxidative stress sensitises mitochondria to opening of the mPTP, an event that leads to cell death [31]. We therefore investigated whether superoxide levels and corresponding antioxidant defence mechanisms were affected by OPA1 dysfunction in D42 iPSC-RGCs. DHE fluorescence was 1.7-fold higher in R445H compared to isogenic control neurons (Fig. 2a). Similarly, MitoSOX fluorescence was 1.2-fold higher in cells harbouring the pathogenic OPA1 variant (Fig. 2b). These results suggest that oxidative stress arises from mitochondrial dysfunction in OPA1-DOA. We also examined antioxidant defence mechanisms by measuring SOD1 protein levels by Western blotting. SOD1 levels (which localises to the cytosol and mitochondrial intermembrane space (IMS)) were not significantly different between genotypes (Fig. 2c & Supplementary Figure S1).

Depolarisation of MMP may result from dysfunction of the ETC, which usually pumps protons into the IMS to sustain the electrochemical gradient that drives ATP synthesis. As there is a bioenergetic defect in DOA iPSC-RGCs [12], we measured MMP in iPSC-RGCs using TMRE, a cationic fluorescent dye that localises to the mitochondrial matrix in response to the potential difference (Fig. 2d). iPSC-RGCs harbouring the R445H variant showed a decrease in TMRE fluorescence compared with isogenic control neurons, suggesting partial depolarisation of MMP (Fig. 2e).

ATP synthase utilises the electrochemical gradient generated by the ETC to synthesise ATP during oxidative phosphorylation [32]. However, if the free energy available from the membrane potential fails to balance the free energy available from the ATP/ADP ratio, the ATP synthase will reverse to act as a proton pumping ATPase, consuming (glycolytic) ATP and maintaining a membrane potential [33]. As such, ATP hydrolysis has been proposed as a mechanism that maintains MMP in bioenergetically compromised neurons and has been described in a range of mitochondrial disease models [34–36]. We therefore tested whether OPA1 dysfunction affects ATP hydrolysis in iPSC-RGCs using TMRE and the ATP synthase inhibitor oligomycin (Fig. 2f). In isogenic control neurons, oligomycin treatment elicited an increase in TMRE fluorescence over time, indicating gradual hyperpolarisation of MMP as expected (Fig. 2g). By contrast, in R445H iPSC-RGCs TMRE fluorescence decayed in response to ATP synthase inhibition (Fig. 2h). At the endpoint of the experiment, TMRE fluorescence was decreased by 43% in R445H compared to isogenic control neurons (Fig. 2i), showing that MMP is partially maintained by ATP hydrolysis in DOA iPSC-RGCs. These findings also highlight ETC dysfunction (i.e. reduced proton pumping by complexes I, III and IV) associated with OPA1 dysfunction in accordance with our previous data [12].

### Altered cytosolic calcium handling in OPA1 R445H iPSC-RGCs in response to ER calcium release

Calcium signalling plays a key role in neuronal function, including regulation of gene expression [37], synaptic function [38], and mitochondrial metabolism [39]. To study calcium signalling dynamics in iPSC-RGCs, calcium uptake into the ER was inhibited with thapsigargin, an irreversible inhibitor of sarcoplasmic/endoplasmic reticulum Ca^2+^-ATPase (SERCA), in calcium-free recording buffer. As this results in depletion of ER stores, reintroduction of calcium into the recording buffer enables the assessment of store-operated calcium entry (SOCE), in which stromal interaction molecules (STIM1/2) and ORAI channel (ORAI1-ORAI3) complexes, and transient receptor potential channels (TRPC1-TRPC7) at the plasma membrane facilitate extracellular calcium influx to the cytosol in an attempt to refill ER calcium stores [40].

**Fig. 3:**
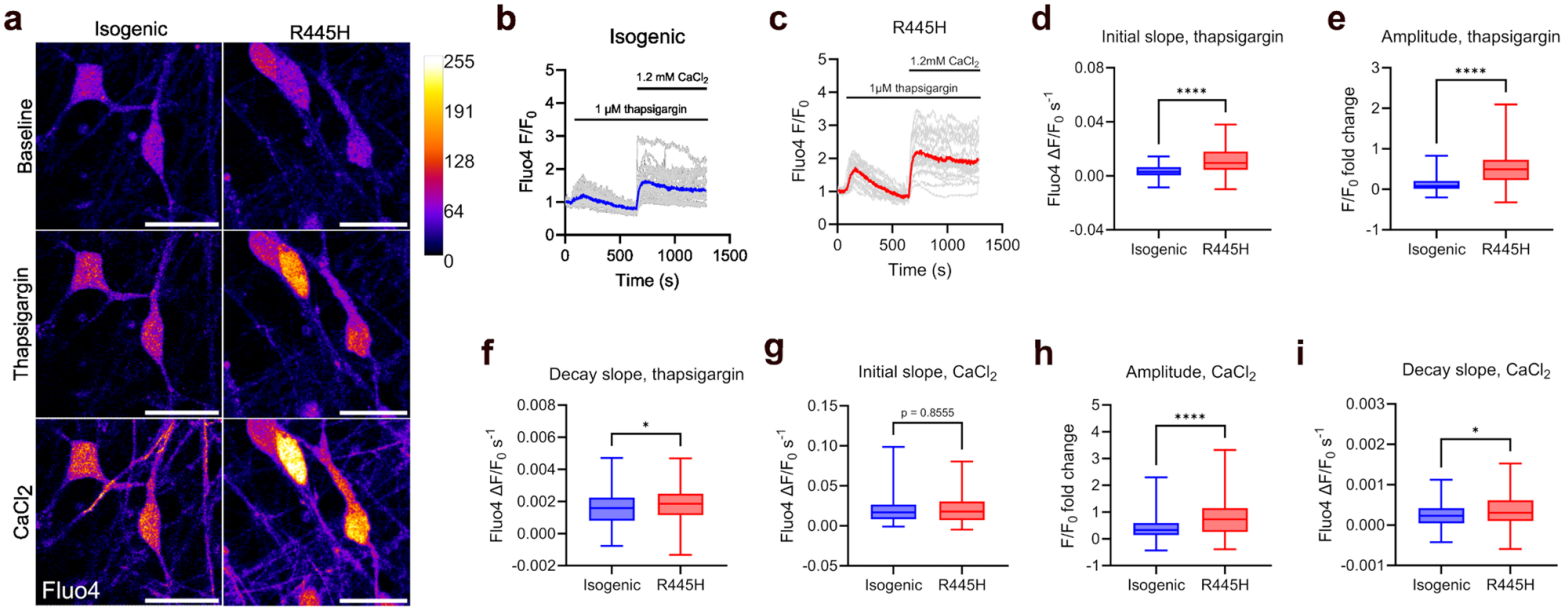
Dysregulation of cytosolic calcium homeostasis in OPA1 R445H iPSC-RGCs in response to ER calcium release. (**a**) Representative live cell confocal images of cytosolic calcium levels in isogenic control and R445H neurons at D42 stained with Fluo4 at baseline, and after treatment with 1.5 µM thapsigargin and 1.2 mM CaCl_2_. Scale bar, 20 μm. (**b** & **c**) Quantification of fold-change in Fluo4 fluorescence intensity over time normalised to baseline values, denoted F_0_. 1.5 µM thapsigargin and 1.2 mM CaCl_2_ were added at the indicated time points. Neurons were maintained in calcium-free recording buffer until addition of CaCl_2_. Grey lines show Fluo4 fluorescence intensity in individual cells from a representative experiment, blue/red lines the mean average normalised Fluo4 fluorescence intensity. (**d**-**i**) Quantification of initial slope, amplitude and decay slope of Fluo4 fluorescence intensity in response to 1.5 µM thapsigargin and 1.2 mM CaCl_2_ in isogenic control R445H and iPSC-RGCs. Isogenic control, *n* = 140; R445H, *n* = 190 cells sampled from three independent differentiations. * = *p* < 0.05; **** = *p* < 0.0001, Mann-Whitney tests. Box plots show median (middle line), 25th-75th percentile (box) and min/max values (whiskers)

After treatment with thapsigargin, R445H iPSC-RGCs displayed an elevated initial slope (2.9-fold increase) and amplitude (3.7-fold increase) of Fluo4 fluorescence compared to isogenic control neurons (Fig. 3a-e). The rate of peak Fluo4 fluorescence recovery to baseline levels was higher in R445H iPSC-RGCs compared to isogenic controls (Fig. 3a-c & f), indicating faster calcium clearance after reaching peak levels. These data suggest that OPA1 dysfunction is associated with altered cytosolic calcium handling upon SERCA blockade and the release of calcium from the ER.

After store depletion, reintroduction of calcium to the recording buffer led to a 1.9-fold elevation of amplitude of Fluo4 fluorescence in R445H iPSC-RGCs compared to isogenic controls (Fig. 3a-c & g & h), indicating higher levels of SOCE associated with OPA1 dysfunction. The rate of Fluo4 fluorescence decay after the Ca^2+^ influx was also increased in R445H iPSC-RGCs compared to isogenic controls (Fig. 3a-c & i), similar to the pattern seen after thapsigargin treatment, indicating faster calcium clearance after reaching peak levels.

### Altered cytosolic calcium handling in OPA1 R445H iPSC-RGCs in response to physiological glutamate challenge

We then tested the hypothesis that OPA1 dysfunction would lead to changes in calcium handling following stimulation with the excitatory neurotransmitter glutamate. Glutamate activates the influx of extracellular calcium into the cytosol, whereby the opening of ionotropic NMDA receptors in particular mediates a significant increase in intracellular calcium levels [41]. Mitochondria act to buffer elevations in cytosolic calcium, utilising the electrochemical gradient generated by the ETC to draw calcium ions into the matrix via the mitochondrial calcium uniporter (MCU) [42].

**Fig. 4:**
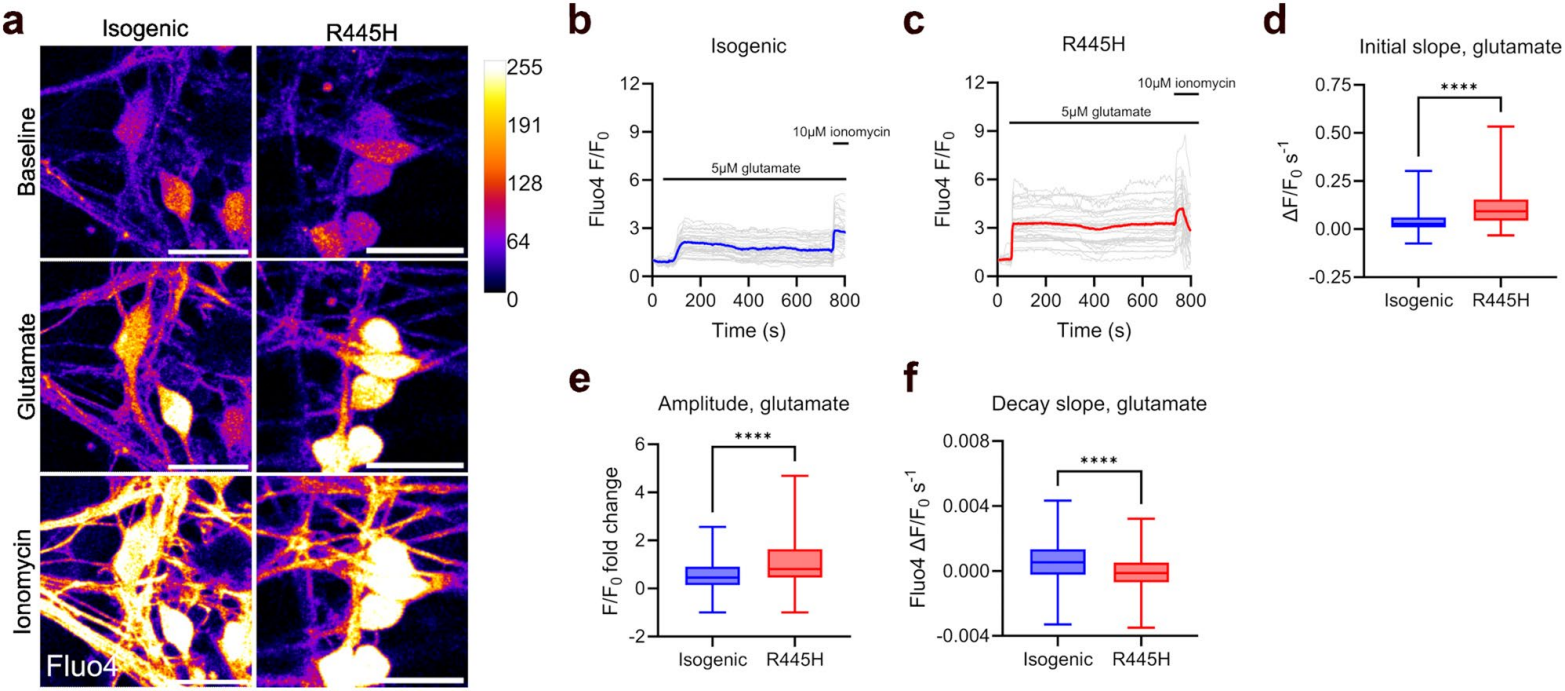
Cytosolic calcium homeostasis is dysregulated in response to the excitatory neurotransmitter glutamate in OPA1 R445H iPSC-RGCs. (**a**) Representative live cell confocal images of cytosolic calcium levels in isogenic control and R445H neurons stained with Fluo4 at baseline, and after stimulation with 5 µM glutamate and 10 µM ionomycin. Scale bar, 20 μm. (**b** & **c**) Quantification of fold-change in Fluo4 fluorescence intensity over time normalised to baseline values, denoted F_0_. 5 µM glutamate and 10 µM ionomycin were added at the indicated time points. Neurons were maintained in magnesium-free recording buffer throughout the experiment. Grey lines show Fluo4 fluorescence intensity in individual cells from a representative experiment, blue/red lines the mean average normalised Fluo4 fluorescence intensity. (**d**-**f**) Quantification of initial slope, amplitude and decay slope of Fluo4 fluorescence intensity in response to 5 µM glutamate in isogenic control R445H and iPSC-RGCs. Isogenic control, *n* = 167; R445H, *n* = 210 cells sampled from three independent differentiations. **** = *p* < 0.0001, Mann-Whitney tests. Box plots show median (middle line), 25th-75th percentile (box) and min/max values (whiskers)

R445H iPSC-RGCs exhibited an elevated initial slope (2.6-fold) and amplitude (1.9-fold) of Fluo4 fluorescence upon glutamate challenge compared to isogenic controls, suggesting that this excitatory pathway may lead to a faster accumulation and higher level of calcium in the cytosol (Fig. 4a-e). The rate of Fluo4 fluorescence decay was lower in R445H compared to isogenic neurons. Whilst isogenic control iPSC-RGCs displayed a gradual decrease in Fluo4 fluorescence after the peak, suggesting restoration of cytosolic calcium homeostasis, R445H neuron Fluo4 fluorescence remained largely at peak levels, suggesting cytosolic calcium homeostasis could not be restored (Fig. 4a-c & f). These data suggest OPA1 dysfunction limits the capacity of human RGCs to restore calcium homeostasis in response to excitotoxic stress. In summary, these data suggest OPA1 dysfunction in iPSC-RGCs leads to perturbations in cytosolic calcium handling in response to SERCA blockade and SOCE, and after treatment with glutamate.

### Increased sensitivity to opening of the mPTP in OPA1 R445H iPSC-RGCs

Mitochondria play a key role in regulating intracellular calcium signalling under normal conditions, but excessive mitochondrial calcium uptake may contribute to pathology by facilitating the opening of the mPTP, a process thought to be exacerbated by oxidative stress [43]. mPTP opening is associated with the collapse of MMP and cessation of ATP synthesis, and is therefore a catastrophic event for neurons that depend mainly on oxidative phosphorylation to meet their energy requirements [31]. To test the threshold for mPTP opening, we challenged R445H and isogenic control iPSC-RGCs with ascending doses of ferutinin, an electrogenic calcium ionophore that increases mitochondrial calcium levels, eventually leading to mitochondrial calcium overload and mPTP opening [34].

**Fig. 5:**
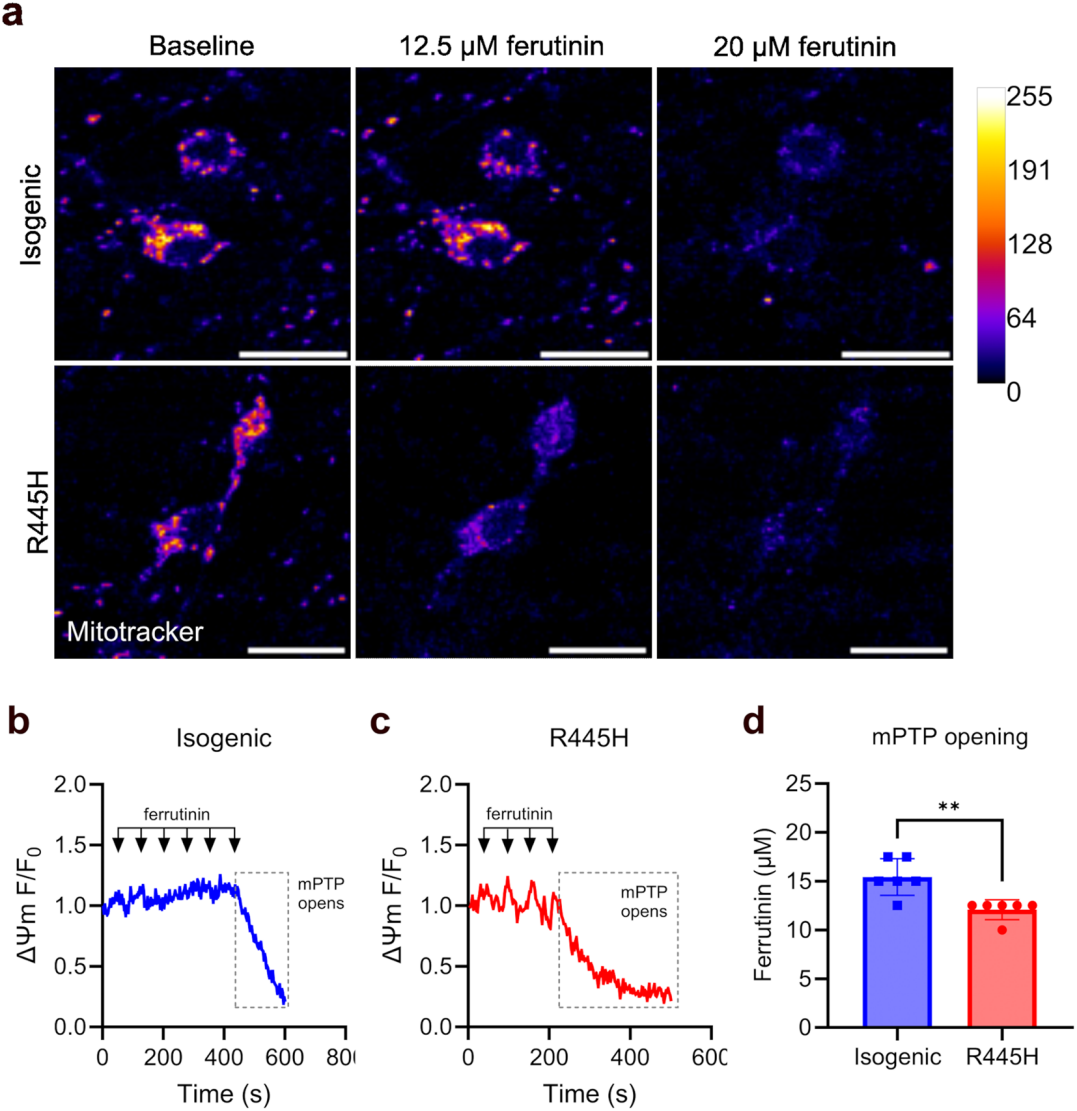
Increased sensitivity to opening of the mPTP in OPA1 iPSC-RGCs in response to mitochondrial calcium overload. (**a**) Representative live cell confocal images of the MMP in Mitotracker-stained isogenic control and R445H iPSC-RGCs at baseline and after treatment with 12.5 µM and 20 µM ferutinin. Scale bar, 20 μm. (**b** & **c**) Quantification of fold-change in Mitotracker fluorescence over time in response to mitochondrial calcium overload in isogenic control and R445H neurons. Fluorescence intensity was normalised to baseline values (F_0_). Ferutinin concentration was increased in 2.5 µM increments at the indicated time points indicated by the arrows. Loss of Mitotracker fluorescence indicates mitochondrial depolarisation due to opening of the mPTP (grey box). (**d**) Quantification of ferutinin concentration required to open the mPTP. Isogenic control/R445H, *n* = 7 experiments (~ 350 cells/genotype) sampled from four independent differentiations. ** = *p* < 0.01, unpaired t-test. Scatter plot shows the mean values +/- SD

The collapse of MMP was measured as a readout for mPTP opening, and the concentration of ferutinin required to induce a rapid loss of MMP was significantly lower in R445H iPSC-RGCs compared to isogenic controls (Fig. 5a-d; R445H, ~ 12.5 µM vs. isogenic, 15.0-17.5 µM ferutinin). These data suggest that the threshold for mPTP opening may be reduced by OPA1 dysfunction in iPSC-RGCs.

## Discussion

The results presented in this study suggest that OPA1 dysfunction driven by a DOA+-associated pathogenic variant, R445H, leads to defects in mitochondrial structure, ETC function, MMP, calcium signalling, and mPTP opening in human RGCs, highlighting pathophysiological mechanisms that could contribute to vision loss in DOA patients.

Consistent with the role of OPA1 in facilitating fusion of the IMM [44], OPA1 neurons displayed fragmented mitochondrial networks in RGC somas and shorter neurite mitochondria compared to isogenic control neurons, data which were corroborated by EM ultrastructural analysis. OPA1 dysfunction was also associated with increased mitochondrial footprint in RGC somas and decreased neurite mitochondrial area, but not a statistically significant change in neurite mitochondria distribution, which may have been mediated by differences in mitochondrial anterograde/retrograde transport [45], or mitochondrial quality control mechanisms [46].

We also detected increased levels of ROS generation, specifically superoxide anions, in OPA1 R445H iPSC-RGCs. These results are in accordance with previous reports of the effect of OPA1 dysfunction in fibroblasts, in which galactose was used in place of glucose in the cell media to force ATP generation by oxidative phosphorylation [47, 48]. A recent study reported increased superoxide levels arising from attenuated OPA1 function in primary vascular cells, with reduced SOD1 protein levels [49], however, we did not detect a statistically significant decrease in SOD1 protein levels in iPSC-RGCs, likely reflecting the different cell types under investigation in the respective studies.

We previously showed bioenergetic defects, in iPSC-RGCs harbouring pathogenic *OPA1* variants suggesting dysfunction of the ETC [12]. In this study, we identified morphological changes in mitochondrial cristae, specifically, the appearance of vesicular cristae in R445H iPSC-RGCs. This structural deficit was associated with a partial depolarisation and maintenance of MMP by ATP hydrolysis in R445H vs. isogenic control iPSC-RGCs, a process that consumes ATP and, therefore, likely reduces the energy supply available for other cellular functions. It remains to be determined whether inhibition of ATP hydrolysis (whilst maintaining ATP synthesis) could be utilised as a potential therapeutic tool in DOA as described in other mitochondrial disease models [35], or if it would accelerate mitochondrial depolarisation and dysfunction.

Cytosolic calcium handling is mediated by a range of mechanisms, including buffering into/release from intracellular stores and influx/efflux mechanisms that operate at the cell surface [37–39]. We observed that neurons with the R445H pathogenic variant exhibited increased calcium release when ER calcium uptake was inhibited, and increased levels of cytosolic calcium after initiation of SOCE. Differences in resting ER calcium levels and/or the activity of the inositol 1,4,5-triphosphate receptors (IP3Rs) and ryanodine receptors (RyRs) which mediate calcium release from the ER could explain these results, however, buffering of cytosolic calcium into the (depolarised) mitochondrial matrix may also have played a role. Given we observed reduced MERC length in OPA1 R445H iPSC-RGCs, it is possible that changes in calcium transfer between mitochondria and ER compartments contribute to calcium deregulation in DOA. OPA1 R445H neurons demonstrated upregulated SOCE which could reflect an attempt to refill ER calcium stores, a pathway that has been described in other neurodegenerative disease models [50–52]. We observed an increased rate of Fluo4 fluorescence decay in R445H compared to corrected iPSC-RGCs following both thapsigargin and CaCl_2_ treatment, suggesting an increase in cytosolic calcium clearance. As this effect is unlikely to be explained by increased ER/mitochondrial calcium uptake, this may reflect the activity of other cytosolic calcium efflux mechanisms, such as Ca^2+^/Na^+^ exchangers (NCX) and/or plasma membrane Ca^2+^ ATPases (PMCA) operating at the cell membrane [53].

Mitochondria serve as buffers to increased cytosolic calcium levels, a process that is dependent on the MMP and MCU activity [42]. As such, the elevation of cytosolic calcium in DOA iPSC-RGCs observed after ER store release and SOCE may be partially explained by reduced uptake of calcium into the mitochondrial matrix, possibly due to MMP depolarisation. In support of this, R445H iPSC-RGCs demonstrated increased Fluo4 fluorescence initial slopes and amplitudes compared to isogenic controls after treatment with glutamate, which might be a consequence of reduced buffering of cytosolic calcium by dysfunctional mitochondria. It is possible that changes in NMDA receptor subunit expression, which have been suggested in previous reports on OPA1 dysfunction [20], could also explain the differences we observed. In contrast to the patterns evident after ER calcium release and SOCE, Fluo4 fluorescence remained elevated in R445H iPSC-RGCs after reaching peak levels. This suggests that calcium clearance is attenuated after glutamate challenge in human RGCs with OPA1 dysfunction, in agreement with previous reports detailing the induction of DCD in Opa1 mouse RGCs after glutamate treatment [19]. In summary, these investigations into calcium handling identified defects associated with OPA1 dysfunction in iPSC-RGCs, in which both the ER and mitochondrial cellular compartments could play a role. In the future it could be important to assess mitochondrial calcium levels specifically, as well as the role that neuronal activity plays in mediating intracellular calcium signalling. Given the metabolic demand placed upon neurons when firing action potentials, it is possible that the observed changes in neuronal activity observed between OPA1 and WT RGCs [54] correlate with the changes in calcium homeostasis observed in this study.

mPTP opening is associated with the collapse of MMP and cessation of ATP synthesis. We challenged cells with ascending doses of ferutinin, an electrogenic calcium ionophore, to induce mitochondrial calcium overload and assayed loss of MMP as a biomarker for mPTP opening. We found that ferutinin induced mPTP formation at lower concentrations in R445H iPSC-RGCs compared to isogenic control neurons. The threshold for mPTP opening is known to decrease when matrix calcium and ROS levels are high [43]. Given that we have identified changes in cytosolic calcium handling and increased oxidative stress in OPA1 R445H iPSC-RGCs, it is possible that OPA1 dysfunction sensitises RGCs to mPTP opening via mitochondrial calcium deregulation and increased superoxide production.

The R445H amino acid substitution in the OPA1 GTPase domain is associated with a DOA+ phenotype and might have some dominant negative activity that could lead to additional OPA1 dysfunction compared to a pure loss of function haploinsufficiency variant. Indeed, we observed greater reductions in mtDNA levels in this OPA1 R445H iPSC-RGCs than in an isogenic heterozygous *OPA1* knock-out line, whereas their bioenergetic deficits were similar [12]. It will be interesting in future to analyse other OPA1 variant iPSC-RGCs to investigate if they show similar, or milder, differences in mitochondrial networks, ROS levels, calcium homeostasis and mPTP opening. DOA primarily affects RGCs, and although our differentiation protocol produces mainly RGC-like neurons [12], it is possible that the neurons we visualised in the live imaging might not be RGCs, but another type of neuron. The introduction by gene editing of an RGC specific fluorescent reporter, such as a *BRN3B*:TdTomato line [55], could potentially enable the direct visualisation of iPSC-RGC cells prior to live imaging, but the reporter would need to be edited into all patient and corrected iPSC lines and might restrict the other fluorophores that could be used, for example TMRE could not be used with TdTomato.

## Conclusions

In summary, this study identified several structural and functional defects associated with a DOA+-associated pathogenic OPA1 variant, R445H, in human RGCs. OPA1 R445H neurons exhibited fragmented mitochondrial networks, oxidative stress and MMP defects. Cytosolic calcium handling was also affected, and the threshold for mPTP opening was reduced. Collectively, these mechanisms may contribute to progressive vision loss in DOA patients.

## Electronic supplementary material

Below is the link to the electronic supplementary material.

Supplementary Material 1

## Data Availability

No datasets were generated or analysed during the current study.

## Abbreviations

ATP: Adenosine triphosphate
CRISPR: Clustered regularly interspaced short palindromic repeats
DCD: Delayed calcium deregulation
DHE: Dihydroethidium
DOA: Dominant optic atrophy
DOA+: Dominant optic atrophy plus
ECL: Enhanced chemiluminescence
ER: Endoplasmic reticulum
ETC: Electron transfer chain
GABA: Gamma aminobutyric acid
GTP: Guanosine triphosphate
HEPES2: (4-(2-hydroxyethyl)-1-piperazinyl)-ethanesulfonic acid
HRP: Horse radish peroxidase
IMM: Inner mitochondrial membrane
IMS: Inter membrane space
iPSC: Induced pluripotent stem cell
IP3R: Inositol 1,4,5-trisphosphate receptor
MCU: Mitochondrial calcium uniporter
MERC: Mitochondria: endoplasmic reticulum contact
MMP: Mitochondrial membrane potential
mtDNA: Mitochondrial DNA
NCX: Sodium calcium exchanger
NCLX: Mitochondrial sodium calcium exchanger
NMDA: N-methyl D-aspartic acid
OCT: Optical coherence tomography
OPA1: Optic atrophy 1
ORAI: Calcium release-activated calcium modulator 1
PBS: Phosphate buffered saline
PMCA: Plasma membrane calcium ATPase
RGC: Retinal ganglion cell
RNFL: Retinal nerve fibre layer
ROI: Region of interest
ROS: Reactive oxygen species
RyR: Ryanodine receptors
SERCA: Sarcoplasmic endoplasmic reticulum
siRNA: Small interfering RNA
SOD1: Superoxide dismutase
SOCE: Store operated calcium entry
STIM: Stromal interaction molecule
TMRE: Tetramethylrhodamine ethyl ester
TRPC: Transient receptor potential channel
TUBB3: β3-tubulin
