# Supplementary material for: Disruption of mitochondrial homeostasis and permeability transition pore opening in OPA1 iPSC-derived retinal ganglion cells
Source: Acta Neuropathol Commun. 2025 Feb 13;13:28. doi: 10.1186/s40478-025-01942-z (PMC11823152; doi:10.1186/s40478-025-01942-z)
Supplement: Supplementary file 1 — Supplementary Material 1 [file 40478_2025_1942_MOESM1_ESM.pdf]

Supplementary material.
Disruption of mitochondrial homeostasis and permeability transition pore opening in OPA1 iPSC-derived retinal ganglion cells
Whitehead et al 2024

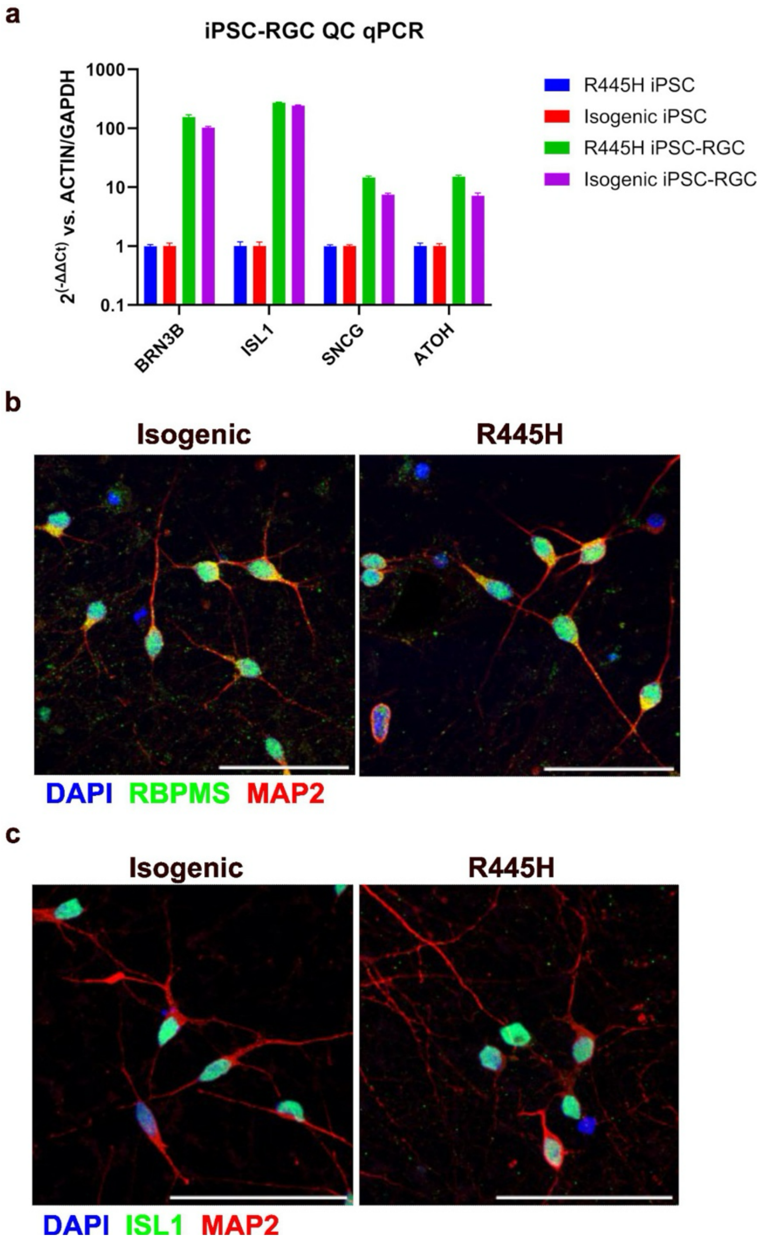

Supplementary Figure S1. RGC marker expression in iPSC-RGCs. (**a**) Representative qPCR for *BRN3B*, *ISL1*, *SCG* and *ATOH* in iPSC (red and blue) and D42 iPSC-RGC (green and purple) for isogenic control (red and purple) and OPA1 R445H (blue and green). (**b** and **c**) Representative immunocytochemistry quality control for differentiations with for RGC markers RBPMS (**b**, green) and ISL1 (**c**, green) neuronal marker MAP2 (red) with DAPI as nuclear stain as Scale bar, 50 μm

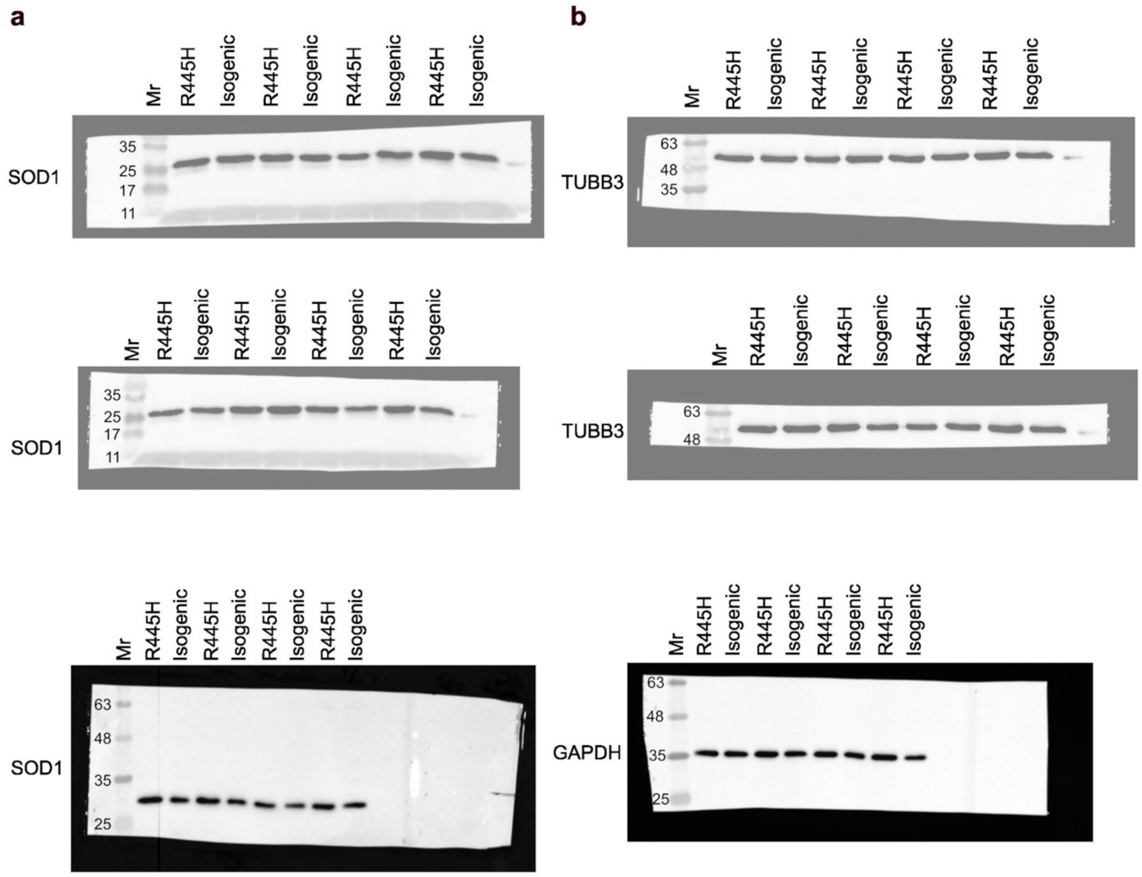

Supplementary Figure S2 (related to Figure 2c). SOD1 levels in OPA1 iPSC-RGCs.

Westerns blots of **a**) SOD1 and **b**) reference proteins (TUJ1 and GAPDH). R445H and isogenic control lanes as indicated. The mobility of molecular weight markers are indicated in lane Mr and their weight indicated on the left (in kDa). Note these are full unmodified blots of cut membranes.

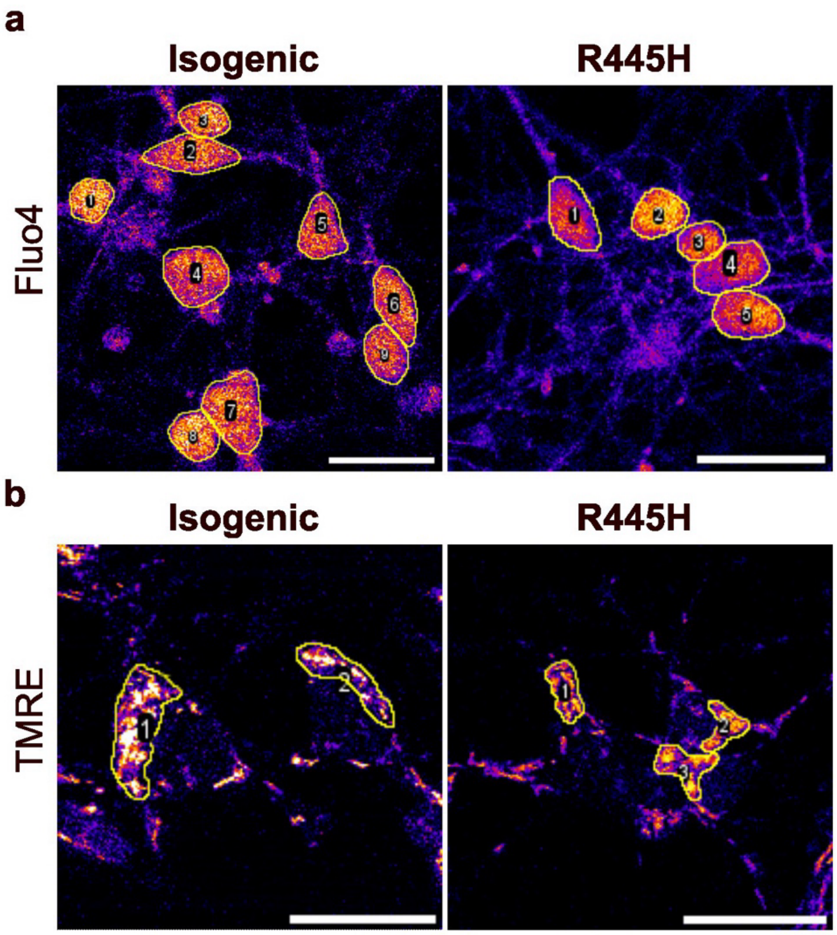

Supplementary Figure S3 (related to Figures 2, 3 and 4). Example regions of interest from live imaging. Example ROIs for Fluo4 (a) and TMRE (b) stained iPSC-RGCs. Scale bar, 20 μm

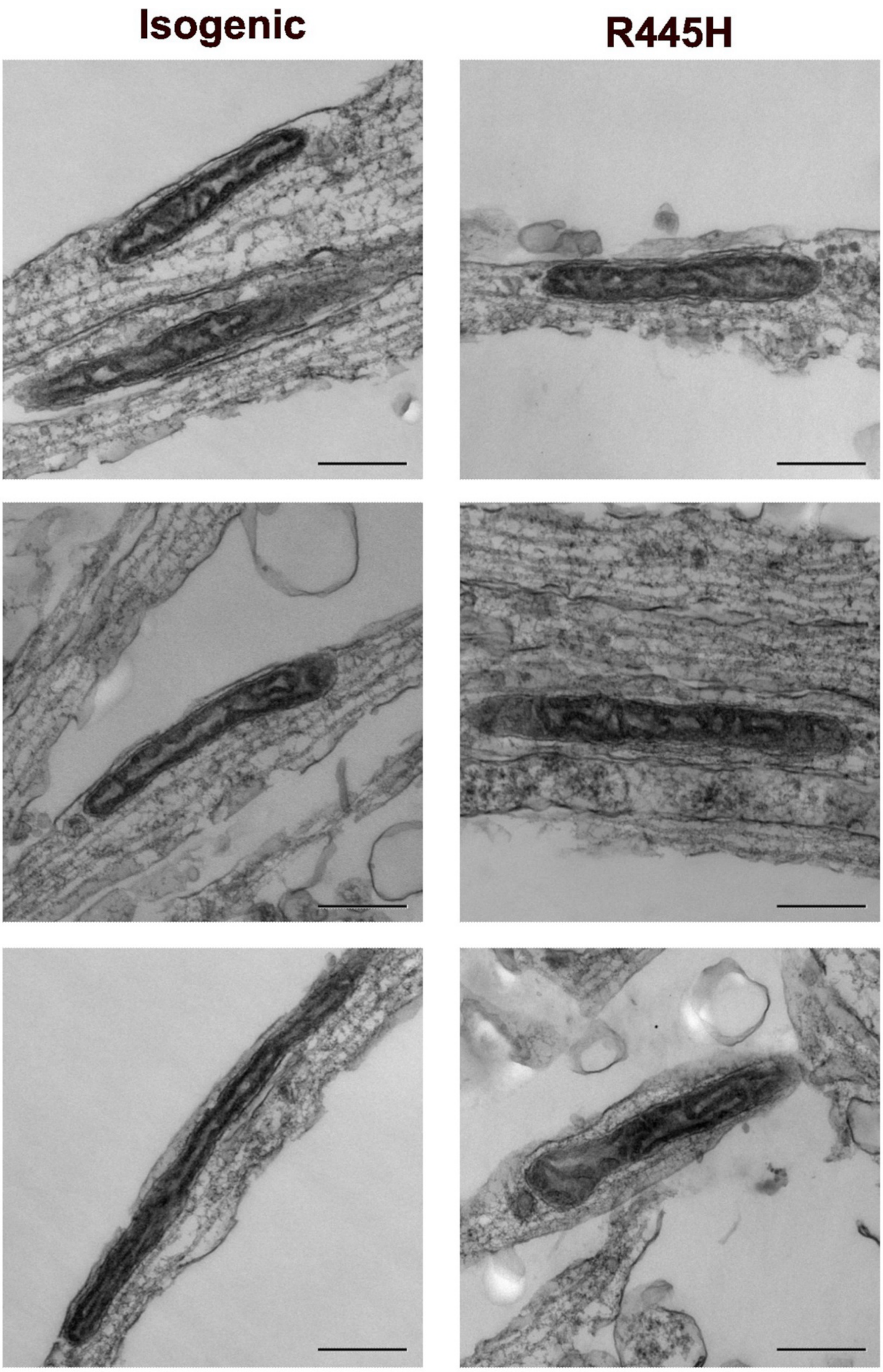

Supplementary Figure S4 (related to Figures 1). Ultrastructure of neurite mitochondria. TEM of OPA1 R445H iPSC-RGC and isogenic corrected control neurites showing mitochondrial morphology. Scale bar, 500 nm
